# Long-term safety and efficacy of sustained eculizumab treatment in patients with paroxysmal nocturnal haemoglobinuria

**DOI:** 10.1111/bjh.12347

**Published:** 2013-04-25

**Authors:** Peter Hillmen, Petra Muus, Alexander Röth, Modupe O Elebute, Antonio M Risitano, Hubert Schrezenmeier, Jeffrey Szer, Paul Browne, Jaroslaw P Maciejewski, Jörg Schubert, Alvaro Urbano-Ispizua, Carlos de Castro, Gérard Socié, Robert A Brodsky

**Affiliations:** 1 St James's University HospitalLeeds, UK; 2 Radboud University Nijmegen Medical CentreNijmegen, The Netherlands; 3 University of Duisburg-EssenEssen, Germany; 4 Department of Haematological Medicine, King's College HospitalLondon, UK; 5 Federico II UniversityNaples, Italy; 6 Institute of Clinical Transfusion Medicine and Immunogenetics, German Red Cross Blood Transfusion Service and Institute of Transfusion Medicine, University of UlmUlm, Germany; 7 Royal Melbourne HospitalMelbourne, Australia; 8 St. James's Hospital, Trinity College DublinDublin, Ireland; 9 Taussig Cancer Center, Cleveland ClinicCleveland, OH, USA; 10 Saarland University Medical SchoolHomburg-Saarland, Germany; 11 Grupo de Trabajo de HPN de la Sociedad Española de Hematología y HemoterapiaBarcelona, Spain; 12 Duke University Medical CenterDurham, NC, USA; 13 Hôpital Saint-Louis and Institut National de la Santé et de la Recherche Médicale (INSERM)Paris, France; 14 Johns Hopkins University School of MedicineBaltimore, MD, USA

**Keywords:** eculizumab, paroxysmal nocturnal haemoglobinuria, phase III, long-term therapy, haemolysis

## Abstract

Paroxysmal nocturnal haemoglobinuria (PNH) is characterized by chronic, uncontrolled complement activation resulting in elevated intravascular haemolysis and morbidities, including fatigue, dyspnoea, abdominal pain, pulmonary hypertension, thrombotic events (TEs) and chronic kidney disease (CKD). The long-term safety and efficacy of eculizumab, a humanized monoclonal antibody that inhibits terminal complement activation, was investigated in 195 patients over 66 months. Four patient deaths were reported, all unrelated to treatment, resulting in a 3-year survival estimate of 97·6%. All patients showed a reduction in lactate dehydrogenase levels, which was sustained over the course of treatment (median reduction of 86·9% at 36 months), reflecting inhibition of chronic haemolysis. TEs decreased by 81·8%, with 96·4% of patients remaining free of TEs. Patients also showed a time-dependent improvement in renal function: 93·1% of patients exhibited improvement or stabilization in CKD score at 36 months. Transfusion independence increased by 90·0% from baseline, with the number of red blood cell units transfused decreasing by 54·7%. Eculizumab was well tolerated, with no evidence of cumulative toxicity and a decreasing occurrence of adverse events over time. Eculizumab has a substantial impact on the symptoms and complications of PNH and results a significant improvement in patient survival.

Paroxysmal nocturnal haemoglobinuria (PNH) is a rare, progressive and life-threatening haematopoietic stem cell disorder. It is characterized by complement-mediated intravascular haemolysis and a prothrombotic state (Brodsky, 2009; Hillmen *et al*, 2010). PNH may be diagnosed in patients of all ages, but the median age is in patients in their early 30s (Socié *et al*, 1996; Nishimura *et al*, 2004). Despite best historical supportive care, including transfusions and anticoagulation management, PNH is fatal in approximately 35% of patients within 5 years of diagnosis (Hillmen *et al*, 1995; Socié *et al*, 1996; Kelly *et al*, 2011).

The disease arises from a somatic mutation in the phosphatidylinositol glycan class A gene (*PIGA*), which disrupts synthesis of glycosylphosphatidylinositol (GPI), a molecule that anchors proteins to cell membranes (Takeda *et al*, 1993). The mutation results in the reduction or complete absence of the GPI-anchored complement regulatory proteins CD55 and CD59, which renders the cells sensitive to complement-mediated destruction (Rother *et al*, 2005; Kelly *et al*, 2009). All blood cells are susceptible to complement-mediated attack, although red blood cells are the most susceptible to lysis (Parker, 1991; Pu & Brodsky, 2011).

Complement activation in PNH is manifested as chronic haemolysis that leads to the release of free haemoglobin and the subsequent depletion of nitric oxide. Consumption of nitric oxide leads to vaso-occlusion and platelet activation and results in the common morbidities seen in PNH, such as fatigue, dyspnoea, recurrent abdominal pain, dysphagia, chest pain and pulmonary hypertension. More importantly, chronic haemolysis renders PNH patients at a greater risk of thrombotic events (TEs), renal insufficiency and other organ damage, and premature mortality. Compared with the general population, patients with PNH have a 62-fold higher risk of a TE (McKeage, 2011) and a six-fold greater risk of chronic kidney disease (CKD) (Hillmen *et al*, 2010). TEs have been reported to account for up to 67% of PNH-related deaths (Hillmen *et al*, 2007), while CKD has been reported to account for 8–18% of disease-related mortality (Clark *et al*, 1981; Nishimura *et al*, 2004).

Historically, supportive care measures used in PNH, such as transfusions, anticoagulation or long-term use of steroids and pain medication, leave patients with a poor quality of life and are associated with adverse events. In addition, supportive care measures do not prevent the occurrence of catastrophic events, such as thromboses and CKD, that have recently been associated with elevated risk of premature mortality (Hillmen *et al*, 2004, 2006, 2007, 2010; Rother *et al*, 2005; Brodsky *et al*, 2008; de Latour *et al*, 2009; Hill *et al*, 2010; Kelly *et al*, 2011).

Eculizumab, a humanized monoclonal antibody, inhibits the terminal complement cascade by binding uniquely to human complement protein C5, thereby inhibiting the formation of pro-inflammatory, prothrombotic C5a, and C5b, with subsequent inhibition of assembly of the membrane attack complex (Hillmen *et al*, 2006; Brodsky *et al*, 2008). Although eculizumab suppresses the terminal complement system, the proximal complement system, responsible for opsonization of microorganisms and clearance of immune complexes, remains intact and fully functional (Rother *et al*, 2007). A series of multinational clinical trials demonstrated that eculizumab therapy is well tolerated, leads to a rapid and clinically significant reduction in intravascular haemolysis and, thus, provides substantial clinical benefits (Hillmen *et al*, 2004, 2006, 2007, 2010; Rother *et al*, 2005; Brodsky *et al*, 2008; Hill *et al*, 2010). By inhibiting terminal complement activation and intravascular haemolysis, treatment with eculizumab significantly reduces TEs (Hillmen *et al*, 2007), transfusion requirements (Hillmen *et al*, 2006) and pulmonary hypertension (Hill *et al*, 2010, 2012) and significantly improves renal function (Hillmen *et al*, 2010), gastrointestinal pain (Hill *et al*, 2005a) and patient quality of life (Hillmen *et al*, 2006).

Clinical benefit occurs rapidly in patients with PNH following the initiation of eculizumab. Here we report on continuous administration of eculizumab (for up to 66 months) and the impact of long-term therapy on clinical outcomes in PNH. In addition, we provide greater insight into the safety profile of long-term eculizumab therapy for PNH.

## Methods

### Study design and patients

**Fig 1:**
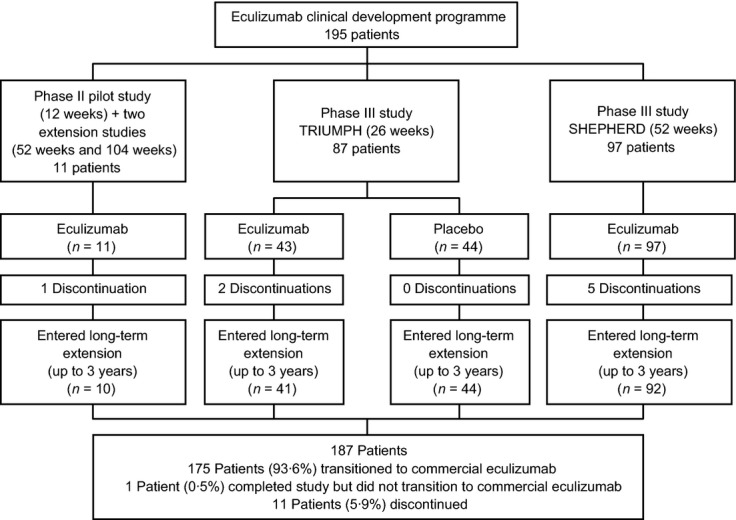
Phase II and III PNH clinical trials overview.

This manuscript reports on the long-term safety and efficacy of eculizumab in 195 patients with haemolytic PNH who had participated in one of three prospective parent trials: the Phase II pilot study (Hillmen *et al*, 2004; Hill *et al*, 2005b) and its extensions, the Phase III TRIUMPH (Transfusion Reduction Efficacy and Safety Clinical Investigation, a Randomized, Multicenter, Double-Blind, Placebo-Controlled, Using Eculizumab in Paroxysmal Nocturnal Haemoglobinuria) study (NCT00122330) (Hillmen *et al*, 2006), or the Phase III SHEPHERD (Safety in Hemolytic PNH Patients Treated With Eculizumab: A Multi-Center Open-Label Research Design) study (NCT00130000) (Hillmen *et al*, 2007; Brodsky *et al*, 2008). At the end of these initial studies, 187 of the 195 patients (95·9%) enrolled in an open-label extension study (Fig 1). All patients had a minimum of 10% PNH red blood cells at enrolment in the parent trials and were vaccinated with a meningococcal vaccine at least 14 d before the first eculizumab infusion in the parent studies. It was recommended that all patients be revaccinated at intervals of between 2·5 and 3 years, with meningococcal vaccine titre assays collected prior to and within 1–2 months of the revaccination. All three parent trials employed the same dosing regimen: 600-mg infusions of eculizumab every week for 4 weeks, followed 1 week later by a single 900-mg dose, and then a maintenance dose of 900 mg every 14 (±2) days until the end of the study. In the extension study patients continued to receive the maintenance dose of eculizumab.

The first clinical investigation of eculizumab was an open-label, 12-week, Phase II pilot study that enrolled 11 patients at two UK study centres, all of whom continued into two extension studies: the first of 52 weeks duration and the second of 104 weeks duration. The positive outcomes from this study (Hillmen *et al*, 2004, 2007) resulted in the initiation of two pivotal phase III studies.

The first pivotal study, TRIUMPH, was a 26-week, double-blind placebo-controlled trial that included 87 patients randomized to either eculizumab (*n* = 43) or placebo (*n* = 44) (Hillmen *et al*, 2006). Patients were required to have had at least four transfusions during the previous 6 months for anaemia or anaemia-related symptoms, platelet counts ≥ 100 × 10^9^/l and lactate dehydrogenase (LDH) levels ≥ 1·5-fold the upper limit of the normal range.

The open-label, single-arm SHEPHERD trial lasted 52 weeks and included 97 patients. The patient eligibility criteria for this study were broadened to include patients with fewer transfusion requirements (as low as one transfusion within 24 months prior to initiating eculizumab treatment) and more severe bone marrow dysfunction (platelet counts as low as 30 × 10^9^/l) (Hillmen *et al*, 2007; Brodsky *et al*, 2008).

The long-term extension study comprised a 104-week treatment period and a 16-week post-treatment follow-up period for any patients who terminated treatment early. Patients who completed the 2-year long-term extension before eculizumab was commercially available or accessible were allowed to continue receiving the investigational product until the drug was approved in their country.

### Study endpoints

Efficacy assessments were performed at least every 2 weeks from the time of initiation of eculizumab therapy in the parent study. Efficacy measures included patient survival, degree of haemolysis (as measured by blood LDH levels), TE incidence rate, mean change from baseline in haemoglobin and the number of units of transfused packed red blood cells (PRBCs) administered. Assessments of renal function were performed over the duration of the study by determining the CKD stage using formulas for estimated glomerular filtration rate (GFR) developed by the Modification of Diet in Renal Disease Study Group (Levey *et al*, 1999; Stevens *et al*, 2006). Specifically, stage 0 was defined as GFR >90 ml/min/1·73 m^2^ with no evidence of kidney damage; stage 1 as GFR >90 ml/min/1·73 m^2^ with evidence of kidney damage; stage 2 as GFR 60–90 ml/min/1·73 m^2^ with evidence of kidney damage (proteinurea assessed by dipstick or abnormal imaging findings); stage 3 as GFR 30–60 ml/min/1·73 m^2^; stage 4 as GFR 15–30 ml/min/1·73 m^2^; and stage 5 as GFR <15 ml/min/1·73 m^2^. The number of transfusion-independent, compared with transfusion-dependent, patients was assessed over time. The baseline period was defined as the 6 months prior to the initiation of eculizumab. At treatment evaluation, transfusion-independent patients were defined as those who did not require a blood transfusion during the previous 6 months, and transfusion-dependent patients had received at least one blood transfusion in the previous 6 months.

Safety assessments included solicited monitoring of adverse events (AEs), clinical laboratory tests and vital signs. AEs were coded using the Medical Dictionary for Regulatory Activities (meddra®) version 6.1, with AEs relating to infection and infection-related serious AEs (SAEs) tabulated separately consistent with the expanded MedDRA preferred terms (http://www.meddramsso.com). In addition, all AEs were reviewed for events that could potentially be related to an infection and classified accordingly. AEs were assessed for severity and classified as mild (events requiring minimal or no treatment and not interfering with the patient's daily activities), moderate (events resulting in a low level of inconvenience or concern with the therapeutic measures and possibly causing some interference with functioning) or severe (events that are usually incapacitating and interrupting usual daily activities that may require systemic drug therapy or other treatment).

An SAE was defined as any event that resulted in death, was immediately life threatening, required inpatient hospitalization or prolongation of existing hospitalization, resulted in persistent or significant disability/incapacity, or was a congenital anomaly/birth defect. Important medical events that did not result in death or require hospitalization and were not life threatening may have been considered SAEs if, based upon appropriate medical judgment, they required medical or surgical intervention to prevent one of the outcomes listed previously.

### Statistical analyses

Baseline was defined as the pre-eculizumab value collected from the parent study, except for those patients in the TRIUMPH study who received placebo where baseline was the value collected at the final visit of this study. Least-squares mean changes from baseline in LDH levels were analysed using a repeated-measure analysis of variance model, including time of treatment, parent study and parent study treatment as fixed effects. The signed-rank test was used to analyse mean changes from baseline in haemoglobin. Change in CKD stage was analysed using the Mantel–Haenszel chi-squared test. An improvement in renal function was defined as a categorical documented reduction in CKD stage, and a worsening in renal function was defined as a categorical documented increase in CKD stage.

Comparisons of the reporting of AEs by patients during the first 26 weeks of treatment with eculizumab and the last 26 weeks of treatment were tested using a one-tailed exact McNemar test using matched-pairs data. For any patient who remained in the study for <52 weeks, the incidence of AEs reported during the first 26 weeks of treatment was compared with the incidence of AEs from 26 weeks + 1 d until the patient's last dose of eculizumab.

Two patient populations were identified for statistical analyses. All patients who had completed at least 26 weeks of randomized treatment in one of the parent studies and had received at least one dose of eculizumab in the extension study were included in the safety population; patients from this population who had no major protocol violations were included in the per-protocol population. All analyses were performed on an intent-to-treat basis. A *P* value of <0.05 was considered to be statistically significant.

## Results

### Study population and baseline characteristics

Baseline characteristics of all 195 patients at the start of the parent trials are shown in Table I. Overall median age was 39·7 years (range: 18–85 years); more than 90% of the patients were Caucasian and 54% were female. The majority of patients (92%) were <65 years of age. Twenty-nine percent of patients had a history of aplastic anaemia and 1·5% had a history of myelodysplastic syndrome.

**Table I:** Baseline patient characteristics

Parameter	Eculizumab (*n* = 195)
Age, years	
Median (range)	39·7 (18·3–85·0)
Mean (SD)	41·3 (14·37)
Median disease duration (range), months	81·5 (12·0–483·8)
Gender, *n* (%)	
Female	106 (54·4)
Male	89 (45·6)
Race, *n* (%)	
Caucasian	176 (90·2)
Black	7 (3·6)
Asian	6 (3·1)
Other	6 (3·1)
History of aplastic anaemia, *n* (%)	56 (28·7)
History of myelodysplastic syndrome, *n* (%)	3 (1·5)
Haemoglobin, g/l	
Mean (SE)	93·7 (1·14)
Range	48·0–143·0
Lactate dehydrogenase, u/l	
(upper limit of normal: 223 U/l)	
Mean (SE)	2293·3 (84·38)
Median	2129
Range	499–10 300

SD, standard deviation; SE, standard error.

The entire period of eculizumab administration across the parent and extension trials was 66 months, although a 36-month cut-off was used for safety and efficacy assessments to ensure that there were a sufficient number of patients for robust statistical analysis. Of the patients included in this 36-month cut-off, the overall median treatment duration was 30·3 months, with a range of 10·0–66·1 months (interquartile range: 26·2–33·1 months). During maintenance dosing, 21 patients (10·8%) deviated from the protocol dosing interval by receiving treatment <12 d apart on at least one occasion. Of the original 195 patients, 19 patients (9·7%) discontinued treatment over a period of 66 months: nine discontinued because of an AE (see ‘Safety’), seven withdrew consent, two were discontinued based on the decision of the investigator and one was noncompliant with the protocol (Fig 1). Eight of the discontinuations (six withdrawn consents and two AEs) occurred during a parent trial, and the remaining 11 discontinuations (seven AEs, two investigator's decision, one withdrawn consent, one non-compliance) occurred during the long-term extension study.

### Intravascular haemolysis

**Fig 2:**
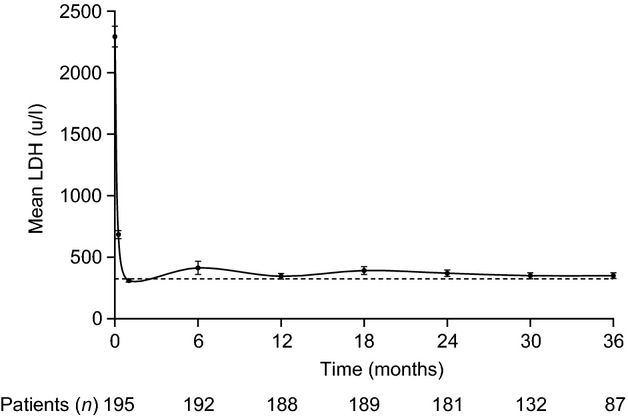
Lactate dehydrogenase level, a marker of intravascular haemolysis, was rapidly and consistently reduced from baseline (*P* < 0·001 at all time points from 1 month) following initiation of eculizumab therapy. Dashed line represents the upper limit of the normal range (103–223 u/l). The decrease in number of patients was due to the transition of patients to commercially available eculizumab. LDH, lactate dehydrogenase.

All patients showed a rapid decrease from baseline in serum LDH, a marker for intravascular haemolysis, which was generally sustained with continued treatment, although throughout the course of the study some patients did have transient increases in LDH. Prior to the first administration of eculizumab, median LDH concentration was 2129 u/l (range: 499–10 300 u/l; upper limit of normal: 223 u/l). One month after eculizumab treatment initiation, median LDH decreased to 263 u/l (range: 93–1208 u/l; *P* < 0·0001; Fig 2), a relative reduction of 87·6%. This decrease was maintained with sustained eculizumab treatment; the median LDH value at 36 months was 279 u/l (range: 88–1417 u/l), a relative reduction from baseline of 86·9%.

Plasma levels of eculizumab and haemolytic activity were reviewed for 140 patients: 43 patients from the TRIUMPH study and 97 patients from the SHEPHERD trial. Following the first dose of eculizumab, trough concentrations <35 μg/ml were observed in 49 of 135 patients (36·3%) with available samples. Overall, 36 of these 49 patients (73·5%) exhibited haemolysis as measured by a validated pharmacodynamic assay (haemolysis >20%). Multiple occurrences of trough eculizumab concentrations <35 μg/ml were observed in 14 of 140 reviewed patients (10·0%). These patients generally had more rapid eculizumab clearance (>0·4 ml/h/kg) or shorter eculizumab half-life (<130 h) than the respective median values of the trial populations as a whole.

None of the patients with breakthrough haemolysis discontinued treatment with eculizumab. In seven of the 14 patients (50·0%), the breakthrough haemolysis was successfully mitigated, as evidenced by reductions in LDH concentrations, by decreasing the eculizumab dosing interval to <14 d. None of these patients experienced SAEs relating to haemolysis. In the whole population, 21 of 195 patients (10·8%) had a median of 22 decreases (range: 3–124) in dosing intervals.

### Haemoglobin levels and transfusion requirements

**Fig 3:**
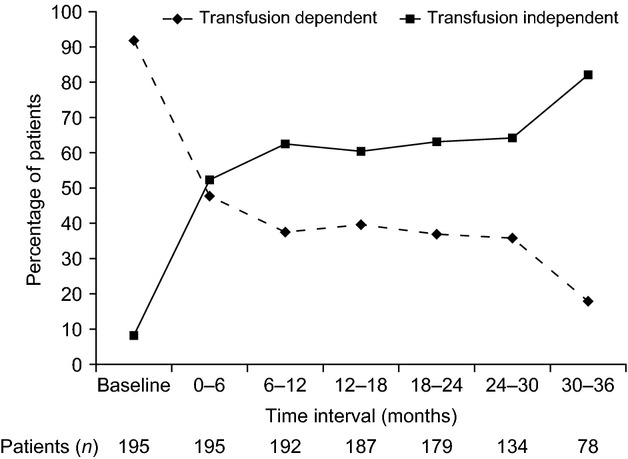
Percentage of transfusion-independent and transfusion-dependent patients over time. Transfusion-independent patients were those who did not require a blood transfusion during the previous 6 months; transfusion-dependent patients had received at least one blood transfusion in the previous 6 months.

Treatment with eculizumab resulted in a steady increase in the serum haemoglobin concentration from baseline (93·7 g/l), including a mean increase from baseline of 5·5 g/l (range: −32·0 to 55·0 g/l) to 99·2 g/l after 2 weeks of treatment and 10·4 g/l (range: −42·0 to 81·0 g/l) to 104·7 g/l by 24 months, which was maintained at 36 months (Table II). The percentage of patients who achieved transfusion independence increased with sustained treatment (Fig 3). The percentage of patients achieving transfusion independence was 82·1% (64 of 78) by the last 6 months of treatment, compared with only 8·2% (16 of 195) in the 6 months prior to the start of treatment, a relative increase of 90.0%. Fourteen of 78 patients (17·9%) continued to require transfusions between months 30 and 36. Despite the need for transfusions, these 14 patients maintained a significant reduction in LDH from baseline levels (*P* < 0·0001), and the number of units of PRBCs transfused over the course of the study significantly decreased from a mean of 11·2 units in the 6 months prior to starting eculizumab to 3·5 units between months 30 and 36 (*P* = 0·0001). The change from baseline in the number of units of PRBCs administered to all patients receiving transfusions over the course of the study is presented in Table II.

**Table II:** Mean number of packed red blood cells transfused and haemoglobin concentrations throughout the study

	Units of packed red blood cell transfused				Haemoglobin (g/l)*			
		
Study period, months	*n*	Mean (SE) [range]	Mean (SE) [range] change from baseline	*P* value	*n*	Mean (SE) [range]	Mean (SE) change from baseline	*P* value
Baseline	164	5·3 (0·22) [1–13]			195	93·7 (1·14) [48–143]		
0–3†	74	4·4 (0·33) [1–13]	−1·5 (0·38) [−8 to 6]	0·0001	195	99·2 (1·05) [64–136]	5·5 (0·96)† [−32 to 55]	<0·0001
3–6	76	4·7 (0·37) [1–19]	−1·5 (0·42) [−9 to 9]	0·0007	193	101·6 (1·15) [65–146]	7·7 (1·41) [−34 to 56]	<0·0001
6–9	61	4·8 (0·40) [2–14]	−1·1 (0·50) [−11 to 9]	0·0293				
9–12	58	4·6 (0·41) [2–18]	−1·7 (0·54) [1–13]	0·0025	190	100·5 (1·23) [36–152]	6·8 (1·51) [−54 to 55]	<0·0001
12–15	62	3·6 (0·29) [1–12]	−2·5 (0·49) [−9 to 10]	0·0001				
15–18	50	4·2 (0·32) [1–11]	−2·0 (0·55) [−11 to 6]	0·0006	189	103·0 (1·21) [63–168]	8·8 (1·45) [−36 to 80]	<0·0001
18–21	50	4·3 (0·51) [1–19]	−2·1 (0·65) [−11 to 15]	0·0022				
21–24	42	4·6 (0·61) [1–17]	−1·4 (0·64) [−11 to 11]	0·0333	178	104·7 (1·23) [65–169]	10·4 (1·59) [−42 to 81]	<0·0001
24–27	37	4·0 (0·49) [1–13]	−2·9 (0·65) [−11 to 4]	<0·0001				
27–30	30	4·4 (0·95) [1–24]	−1·5 (0·98) [−9 to 18]	0·1298	135	106·7 (1·44) [66–159]	11·1 (1·86) [−30 to 71]	<0·0001
30–33	10	3·1 (0·55) [1–7]	−3·0 (0·65) [−6 to 1]	0·0013				
33–36	7	2·4 (0·30) [2–4]	−3·6 (1·13) [−10 to −1]	0·0196	86	105·6 (1·79) [64–146]	9·5 (2·20) [−31 to 68]	<0·0001

SE, standard error.

* Haemoglobin assessment at the end of corresponding study period.

† Haemoglobin assessment occurred 2 weeks after treatment initiation.

### Thrombotic events

Prior to initiation of eculizumab, 63 of 195 patients (32·3%) experienced 124 TEs, both venous and arterial, over a total of 1683 patient-years. These TEs included deep-vein thrombosis, hepatic vein (Budd-Chiari syndrome), cerebrovascular accident, mesenteric thrombosis and pulmonary embolism. The percentage of patients free from TEs increased from 67·7% before treatment to 96·4% during treatment: seven patients (3·6%) reported a total of 10 TEs over 467·1 patient-years. Of these seven patients, five had a history of TEs prior to starting treatment with eculizumab and four were receiving concomitant anticoagulation therapy (including warfarin and low-molecular-weight heparins). Of the 10 TEs reported during eculizumab treatment, five were reported as thrombophlebitis/deep vein thrombosis (three events occurred in the same patient), one as deep vein thrombosis, one as retinal vein thrombosis and one as a possible thrombosis in a fistula. The remaining patient experienced portal vein thrombosis and splenic infarcts. The median time from the first dose of eculizumab to the first TE was 646 d (approximately 21 months), with a range of 170–876 d (approximately 5·6–29·0 months).

A time-matched analysis showed that in the 467·1 patient-years prior to eculizumab treatment, there were a total of 52 TEs reported in 25 patients. The administration of eculizumab reduced the TE incidence rate from 11·13 events per 100 patient-years to 2·14 events per 100 patient-years, a relative reduction of 81·8% (*P* < 0·0005).

Overall, 98 patients (50·3%) were treated with anticoagulants prior to or coincident with eculizumab: 11 patients before the start of the study, 84 patients both before and during treatment with eculizumab and three patients during the study following a TE. Of the 95 patients who were treated with an anticoagulant prior to the start of the study, 58 (61·1%) experienced at least one TE before receiving eculizumab. Eleven patients, six of whom had a prior history of TEs, discontinued anticoagulant therapy while receiving eculizumab. None of these 11 patients experienced a TE after discontinuation of anticoagulant therapy.

### Chronic kidney disease

Chronic kidney disease stages (National Kidney Foundation, 2002) were assessed following each 6-month eculizumab treatment period. Previous reports on this patient population have demonstrated that prior to eculizumab treatment, 64% of patients had evidence of CKD (Hillmen *et al*, 2010). There was a time-dependent improvement in renal function. The percentage of patients showing improvement, worsening or no change in CKD was 25·4%, 6·1% and 68·5% respectively, at 6 months compared with 44·8%, 6·9% and 48·3% respectively, at 36 months. Overall, following 36 months of treatment with eculizumab, 93·1% of patients showed either an improvement or stabilization in CKD, and patients were significantly more likely to experience an improvement than a worsening in renal function (*P* = 0·015).

### Patient survival

**Fig 4:**
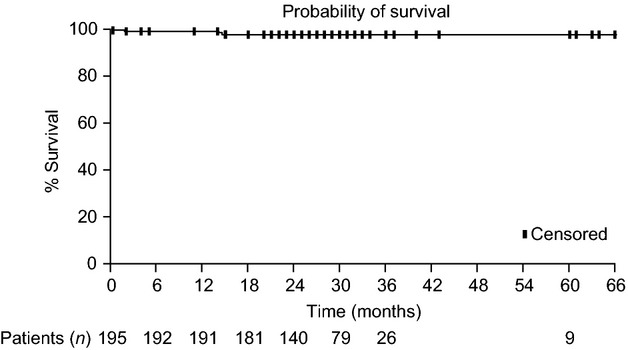
Long-term survival with eculizumab therapy.

Four of the 195 patients died while on eculizumab treatment; the Kaplan–Meier estimate of patient survival at 36 months was 97·6% [95% confidence interval (CI), 93·7–99·1%], which was sustained out to 66 months (Fig 4). Mean duration of therapy with eculizumab was 399 d (range: 60–720 d). Three deaths were caused by concomitant conditions: chronic myelomonocytic leukaemia in a 71-year-old patient who had a history of myelodysplasia and died after 208 d of eculizumab therapy; metastases of pre-existing stomach adenocarcinoma in a 63-year-old patient who died after 609 d of therapy; and cerebral herniation in a 31-year-old patient who died after 60 d of therapy. During in-patient care for a pulmonary embolism, following a series of hospitalizations for a disc prolapse, pneumonia and renal failure, the patient started hyperventilating with his pupils becoming dilated. A computerized tomography scan of the brain revealed a marked left hemorrhagic cerebral arterial media infarct with clear signs of cerebral herniation and the start of cerebrospinal fluid block. The last assessment of LDH levels, almost 4 weeks prior to the event, were below the patient's chronic baseline haemolytic state, leading the investigator to conclude that no serious haemolysis was present at that time.

The fourth patient, a 35-year-old with unconfirmed aplastic anaemia who had received therapy for 720 d, experienced an arteriovenous thrombosis of the small bowel. The investigator decided to discontinue treatment with eculizumab because of the patient's aplastic anaemia; the patient experienced the TE 19 d after the last eculizumab dose, and died with clinical symptoms of sepsis following resection of the small intestine. Further details relating to this patient have been previously reported (van Bijnen *et al*, 2012). None of the deaths were considered related to treatment with eculizumab.

### Safety

Results from this study indicated that eculizumab was well tolerated by patients with PNH. Table III lists AEs that were reported in at least 10% of patients, regardless of relationship to treatment. The most frequently reported AEs were headache, nasopharyngitis and upper respiratory tract infection, each reported in more than 40% of patients. The majority of AEs (91·3%) were mild to moderate in severity.

**Table III:** Treatment-emergent adverse events reported in ≥10% of patients

Adverse event	Number (%) of patients (*n* = 195)
Patients reporting at least one adverse event	194 (99·5)
Headache	107 (54·9)
Nasopharyngitis	97 (49·7)
Upper respiratory tract infection	80 (41·0)
Diarrhoea	68 (34·9)
Nausea	63 (32·3)
Vomiting	50 (25·6)
Back pain	48 (24·6)
Abdominal pain	43 (22·1)
Arthralgia	43 (22·1)
Oropharyngeal pain	42 (21·5)
Pyrexia	40 (20·5)
Cough	39 (20·0)
Dizziness	39 (20·0)
Pain in extremity	39 (20·0)
Influenza-like illness	34 (17·4)
Urinary tract infection	33 (16·9)
Viral infection	30 (15·4)
Constipation	29 (14·9)
Contusion	29 (14·9)
Myalgia	29 (14·9)
Fatigue	25 (12·8)
Abdominal pain upper	24 (12·3)
Insomnia	23 (11·8)
Sinusitis	23 (11·8)
Episataxis	21 (10·8)
Oedema peripheral	20 (10·3)
Pruritus	20 (10·3)
Rash	20 (10·3)

Serious AEs were reported in 75 of the 195 patients (38·5%), with some of the most frequently reported serious AEs, such as haemolysis, abdominal pain and anaemia, typically seen in patients with PNH. These events generally occurred at times of biological stress, most typically in conjunction with an infection. Other serious AEs reported by at least 2% of patients were pyrexia (4·6%) and viral infection (3·1%). Cholecystectomies were reported in six of the 195 patients (3·1%), and one patient developed chronic myelomonocytic leukaemia, resulting in the death of the patient.

Of the 19 patients who discontinued treatment, five (26·3%) discontinued as a result of a nonfatal AE. Two patients became pregnant, one patient developed myelodysplastic syndrome, one patient had meningococcal sepsis (see Table IV) and one patient had, in the opinion of the investigator, a worsening of PNH. This patient suffered from diabetes and had persistent anaemia and impaired renal function, but without reticulocytosis. Of note, three of these 19 patients (15·8%) experienced a TE within 8 weeks of their last dose of eculizumab, with clinical details for one of these patients having been previously described (van Bijnen *et al*, 2012). In the two patients who became pregnant, eculizumab was received for the first 4 and 5 weeks of gestation. Both pregnancies were without complication and resulted in the delivery of healthy babies without any adverse effects from eculizumab treatment (Kelly *et al*, 2010).

**Table IV:** Serious infection-related treatment-emergent adverse events reported during eculizumab therapy (*n* = 195)

Adverse event	Number (%) of patients	Mean onset (days)*
Pyrexia	9 (4·6)	446
Viral infection	6 (3·1)	477
Lower respiratory tract infection	3 (1·5)	833
Urinary tract infection	3 (1·5)	878
Cellulitis	2 (1·0)	235
Meningococcal sepsis	2 (1·0)†	385
Pneumonia	2 (1·0)	456
Respiratory tract infection	2 (1·0)	664
Sepsis	2 (1·0)	604
Septic shock	2 (1·0)	312
Viral gastroenteritis	2 (1·0)	419

* Mean interval between date of first eculizumab dose until the adverse event onset date.

† One patient discontinued treatment.

Three of the patients included in this study had a history of myelodysplastic syndrome (MDS) prior to treatment with eculizumab. At the time of last follow-up, one patient had spontaneous resolution of the disorder 6 years after onset, one patient was alive and had transitioned to commercially available eculizumab and, as mentioned previously, one patient developed chronic myelomonocytic leukaemia and subsequently died. A fourth patient was diagnosed with MDS during treatment with eculizumab and was withdrawn from the study prior to receiving a bone marrow transplantation; this patient did not show any signs of serious haemolysis in the 12 weeks of follow-up following discontinuation of eculizumab.

Forty patients (20·5%) reported a total of 67 serious infection-related treatment-emergent AEs, none of which were fatal. Table IV presents all serious infection-related events reported in two or more patients. The most frequently reported events were pyrexia (reported in nine patients) and viral infection (reported in six patients). There was one case of staphylococcal infection, which was successfully treated with cefazolin sodium and two doses of vancomycin, although the patient subsequently withdrew from the study.

Two cases of meningococcal sepsis were reported during treatment, an infection rate of 0·42 per 100 patient-years. One was a serotype B infection that occurred in a 24-year-old male patient 353 d after the initiation of eculizumab. This patient had received a quradivalent vaccine against serotypes A, C, W135 and Y. The infection was successfully treated with several antibiotics, including imipenem, vancomycin, ceftriaxone and penicillin; the range of antibiotics used was because of bacterial sensitivity and the resistance pattern in the local hospital flora. This patient remained in the study and continued to receive eculizumab. The other case occurred in a 54-year-old female patient 416 d after eculizumab treatment was initiated and was due to serotype Y or W135 (further serotyping was not possible in this patient's country). This patient had been vaccinated against serotypes A and C. This infection was successfully treated with meropenem, vancomycin, ceftriaxone and ciprofloxacin, although the patient discontinued treatment on the advice of the investigator. Of note, neither of the patients with meningococcal sepsis had received vaccination against the specific strain of their infection, at the time of the infections serum bactericidal antibody values were within an appropriate range and both infections resolved with treatment and without sequelae. In neither case was the meningococceal infection due to failure of the vaccine.

All AEs coded as pyrexia were considered related to infection even if an identifiable infection was not diagnosed at the time of the fever. Overall, there were 38 events of pyrexia reported in 22 patients, the majority of which (26 of 38; 68·4%) were mild in severity, whereas only three of 38 events (7·9%) were considered severe. As previously mentioned, nine patients (4·6%) had pyrexia events reported as SAEs, which included all the events considered to be severe. All events of pyrexia resolved without sequelae.

Adverse events related to treatment infusion were reported in 71 patients (36·4%). Peripheral oedema, pruritus and rash were the most frequently reported AEs of this type, each occurring in a total of 20 patients (10·3%). None of the AEs associated with treatment infusion resulted in the discontinuation of eculizumab.

In order to investigate if long-term treatment with eculizumab increased the incidence of any individual or category of AE or whether eculizumab was associated with cumulative toxicity, the incidence of AEs (irrespective of relation to treatment) reported during the first 26 weeks of eculizumab treatment was compared with the last 26 weeks of treatment (Table V). Overall, significantly fewer patients reported at least one AE in the last 26 weeks of treatment (*n* = 145) than in the first 26 weeks of treatment (*n* = 189; *P* < 0·001), and no individual AE was reported by a statistically significantly greater number of patients during the last 26 weeks than during the first 26 weeks of treatment with eculizumab. Although this analysis included patients who had a total treatment duration of <52 weeks, the mean (median) observation times of 26·00 (26·0) and 25·86 (26·0) weeks for the first and last observation periods respectively, indicated that the reduction in number of AEs seen in the last 26 weeks of therapy was not due to the reporting of AEs over a shorter observation time.

**Table V:** Adverse events reported at a significantly different incidence in the first and last 26 weeks of treatment with eculizumab*

	Number of patients		
		
Adverse event	First 26 weeks	Last 26 weeks	*P* value†
Any event	189	145	<0·001
Headache	87	20	<0·001
Nasopharyngitis	47	32	0·029
Nausea	33	21	0·029
Constipation	14	3	0·006
Back pain	20	9	0·031
Arthralgia	17	8	0·032
Muscle spasms	9	0	0·002
Dizziness	17	8	0·039
Pyrexia	18	9	0·032
Epistaxis	12	4	0·011
Fatigue	12	4	0·029
Insomnia	10	2	0·019
Respiratory tract infection	7	0	0·008
Oral herpes	8	1	0·020
Chest pain	6	1	0·031
Haematoma	6	1	0·031
Lethargy	5	0	0·031
Toothache	5	0	0·031

* For patients who received eculizumab for a total of <52 weeks, the incidence of adverse events in the first 26 weeks was compared with the period from the start of the 27th week until the last dose of treatment. Only events for which there was a statistically significant difference between the treatment periods are reported.

† *P* values determined from one-tailed exact McNemar test for matched-pairs data.

Assessments of clinical laboratory tests and vital signs did not reveal any consistent or clinically significant mean changes or any obvious treatment effect on these parameters. There were small increases in direct bilirubin concentrations, although median levels of total bilirubin remained essentially unchanged.

## Discussion

The results from this study demonstrate the long-term safety and efficacy of chronic eculizumab treatment in a large cohort of patients with PNH (*n* = 195). The median eculizumab treatment duration was 30·3 months, with a maximum duration of 66 months. Inhibition of terminal complement activity with eculizumab has previously been reported to rapidly and significantly reduce chronic haemolysis, leading to reductions in transfusion requirements (Hillmen *et al*, 2006) and TEs (Hillmen *et al*, 2007), as well as improvements in renal function (Hillmen *et al*, 2010). The current study shows that these results are sustained with long-term treatment.

Thrombotic events are a severe and life-threatening complication of PNH and are reportedly the most frequent cause of death in patients with PNH (Hillmen *et al*, 2007). TE has been shown to be a significant risk factor for early mortality (relative risk: 15·4; 95% CI, 9·3–25·4; *P* < 0·001) in untreated patients (de Latour *et al*, 2008). Furthermore, following the occurrence of a first TE, there is a greater risk of subsequent TEs, which places patients with a history of TE at a heightened risk of premature mortality (Nishimura *et al*, 2004; de Latour *et al*, 2008). Given that TE in PNH patients appears to be related to severe and ongoing uncontrolled terminal complement activation, management of TE is difficult in PNH patients, as many continue to report TE despite prophylactic use of anticoagulation therapies (Moyo *et al*, 2004; de Latour *et al*, 2008; Kelly *et al*, 2011). We observed an 81·8% reduction in the incidence of TEs with long-term eculizumab treatment. Eighty-four patients in this study received concomitant anticoagulant therapy, which was discontinued in 11 patients. Despite six of these 11 patients having a history of TE, none of them experienced a TE following withdrawal of anticoagulation therapy while receiving eculizumab alone. This experience is consistent with other reports (Emadi & Brodsky, 2009; Kelly *et al*, 2011).

Thrombotic events were observed in 16% (3 of 19) of patients who discontinued treatment with eculizumab, all within 8 weeks of taking their last dose. This finding highlights the importance of adherence to eculizumab treatment at the recommended dose and schedule over the long term, as continuous therapy is paramount in maintaining sustained inhibition of the underlying uncontrolled terminal complement activation. If discontinuation of eculizumab therapy becomes necessary, the potential risk of thrombosis as well as possible preventive measures should be considered.

Chronic kidney disease in PNH patients is caused by several factors, including chronic exposure to free haemoglobin, which increases renal accumulation of haemosiderin, tubulointerstitial inflammation and kidney damage (Nath *et al*, 2001). Renal impairment is known to progress over time (Hillmen *et al*, 2010) and is associated with an increase in mortality (Kim *et al*, 2011). Continued improvement in CKD was observed with sustained eculizumab treatment, with 44·8% of patients showing improvement after 36 months of treatment, compared with 24·4% showing improvement in CKD after only 6 months of treatment. The improvement in renal function in all nine patients with early-stage CKD (stage 1–2) over 36 months suggests that patients treated with eculizumab at earlier stages of kidney damage have a greater likelihood of improvement in renal function, and there is evidence that sustained long-term therapy with eculizumab can reverse early stage renal damage sustained prior to treatment initiation. These are unique findings in a patient population known to be at risk for progressive decline in renal function (Hillmen *et al*, 2010), and they suggest that eculizumab may have a protective role that can lead to improvement in, or less progression of, renal disease.

Reduction in the incidence of TE and CKD, the two major causes of death in PNH, by administration of eculizumab would be expected to lead to improved patient survival. In the current prospective, multinational study in a large cohort of 195 patients, we demonstrated a patient survival of 97·6% (95% CI, 93·7%–99·1%) at 3 years, which was maintained over 66 months of eculizumab treatment. This result is similar to the 5-year survival rate of 95·5% observed in a single-centre, retrospective analysis of 79 patients (Kelly *et al*, 2011). These survival rates seen with eculizumab therapy are a substantial improvement over historical 5-year survival rates of approximately 65% in PNH patients with evidence of haemolysis (Hillmen *et al*, 1995; Kelly *et al*, 2011) and are similar to survival rates of age- and sex-matched healthy subjects (Kelly *et al*, 2011). These results further emphasize the role of uncontrolled complement activation in the pathophysiology of PNH-related mortality.

Long-term treatment with eculizumab also resulted in sustained haematological improvement in patients. Transfusion independence was achieved by 52% (102 of 195) of patients within the first 6 months of treatment, which increased to 82% by the last 6 months of treatment. The number of units of PRBCs transfused was reduced by 55% over the 36-month period. In addition, there was a continuous improvement in haemoglobin levels, which was achieved despite the reduction in transfusions.

The standard eculizumab maintenance dose of 900 mg every 14 ± 2 d was sufficient to maintain inhibition of haemolysis in 89% of patients. In the 21 patients who did report breakthrough haemolysis, shortening the dosing interval to <14 d was sufficient to maintain inhibition of haemolysis, and none of these patients discontinued treatment because of a lack of therapeutic effect. It is important that patients be monitored for any signs or symptoms of breakthrough haemolysis so that the dosing can be adjusted accordingly or, in cases of persistent breakthroughs, to ensure the patient has not developed human anti-human antibodies (HAHA) or neutralizing antibodies to eculizumab. Subsequent to the end of the study, a more sensitive analysis became available for the identification of HAHA in patients receiving eculizumab. With this new analysis, two of the patients in the long-term extension study were found to have low positive values for neutralizing antibodies, which exceeded the prespecified threshold for the *in vitro* assay (data not shown). This finding had no impact on the clinical response to eculizumab therapy or on the pharmacokinetic and pharmacodynamic effects of eculizumab, which continued to block complement activity.

Eculizumab was well tolerated over the long term and demonstrated a positive safety profile. Although nearly all patients reported at least one AE, this is to be expected considering the duration of the study; importantly, discontinuation from treatment due to a nonfatal AE was seen in only five patients (2·6%) over the entire period of study. With no comparator group in this open-label extension, we compared the AE rate of the early treatment group with that of the later treatment group to understand the risk of long-term eculizumab treatment. The probability of a patient experiencing one or more AEs was significantly lower in the last 6 months of the eculizumab treatment period than in the first 6 months of therapy. This suggests that there is no cumulative toxicity with long-term administration of eculizumab and that the risk–benefit profile of eculizumab improves with extended use. Serious infection-related AEs were observed throughout the study, although there was no evidence that their occurrence was related to time on eculizumab treatment.

There is an increased incidence of infections with *N. meningitidis* in individuals with genetic deficiencies of C5–C9 (Figueroa & Densen, 1991). Thus, blocking terminal complement activity with eculizumab increases a patient's susceptibility to meningococcal infections. Two cases of meningococcal sepsis were reported during the approximately 467 patient-years of exposure to eculizumab. Both patients were successfully treated without sequelae. Patient education on the signs and symptoms of potential meningococcal infections remains important to ensure that any infections are identified as quickly as possible. This is particularly crucial outside of a closely monitored clinical study, where continuous vigilance and proactive antibiotic treatment are essential to avoid a potentially fatal infection (http://soliris.net/sites/default/files/assets/soliris_pi.pdf).

In conclusion, long-term treatment with eculizumab resulted in sustained improvement in patient outcomes by rapidly reducing haemolysis and significantly reducing the frequency of severe and life-threatening morbidities, such as TEs and CKD, and thus, improving patient survival. There was evidence that eculizumab improved kidney function over time and also provided sustained protection against further renal damage, particularly when administered early and prior to advanced renal impairment. Eculizumab therapy was well tolerated with a favourable safety profile; very few patients discontinued treatment during long-term therapy. Over the course of this study, survival rates were high and consistent with the improvements observed in another overlapping cohort of patients in whom survival was seen to be similar to age- and sex-matched normal control subjects (Kelly *et al*, 2011). The International PNH Registry (www.pnhregistry.com) is providing further opportunities to continue studying and understanding the long-term benefits of eculizumab in patients with PNH outside the clinical trial setting.
